# Unusual case of *Myroides odoratimimus* infection in a cancer patient: addressing its antimicrobial resistance mechanisms and review of the literature

**DOI:** 10.3389/fcimb.2026.1647777

**Published:** 2026-04-30

**Authors:** Lingying Fan, Yiping Chen

**Affiliations:** Department of Clinical Lab, West China School of Public Health and West China Fourth Hospital, Sichuan University, Chengdu, China

**Keywords:** antibiotic resistance gene, antibiotic susceptibility testing, multidrug resistance, *Myroides odoratimimus*, pH value, urinary tract infection

## Abstract

**Introduction:**

*Myroides odoratimimus* is a Gram-negative opportunistic pathogen primarily affecting immunocompromised individuals. However, its resistance characteristics and therapeutic response in cancer patients remain poorly defined. This study investigated the antimicrobial resistance profile of a urinary *M. odoratimimus* isolate from a gastric cancer patient and evaluated the influence of environmental pH on antibiotic efficacy.

**Methods:**

The isolate was identified using the VITEK 2-Compact system, matrix-assisted laser desorption/ionization time-of-flight mass spectrometry (MALDI-TOF MS), and 16S ribosomal ribonucleic acid (16S rRNA) sequencing. Antimicrobial susceptibility testing against 24 antibiotics was performed using the VITEK 2 system according to Clinical and Laboratory Standards Institute (CLSI) M100 (Ed33) guidelines. Resistance genes were detected by polymerase chain reaction (PCR) and sequencing. The impact of pH (5.5–8.5) on antibiotic activity was assessed using disk diffusion. *In vivo* efficacy of minocycline was evaluated in a murine urinary tract infection model with controlled urine pH modulation.

**Results:**

The isolate showed 100% sequence identity with *M. odoratimimus* PR63069 and exhibited multidrug resistance, remaining susceptible only to minocycline. Molecular analysis confirmed the presence of the β-lactamase gene (blaMUS-1). Tigecycline and minocycline demonstrated optimal activity under neutral conditions (pH 7.3), whereas piperacillin–tazobactam showed relatively improved activity at alkaline pH. *In vivo*, urinary alkalization (pH 8.5) significantly enhanced the antibacterial efficacy of minocycline.

**Conclusion:**

*M. odoratimimus* isolated from a gastric cancer patient demonstrated multidrug resistance mediated by blaMUS-1. Antibiotic activity varied with pH, and urine alkalinization improved minocycline efficacy, suggesting potential strategies for clinical management of Myroides infections.

## 1 Introduction

*Myroides odoratimimus* (*M. odoratimimus*) is an opportunistic environmental pathogen classified as a Gram-negative, non-fermentative, rod-shaped bacterium. Although widely distributed in natural environments, it is not considered a component of the normal human microbiota. Human infections caused by *M. odoratimimus* are uncommon and predominantly occur in immunocompromised individuals. Nevertheless, sporadic outbreaks and fatal cases have been documented (Tran and Juergens, 2023; Ogiso-Tanaka et al., 2022). In recent years, an increasing number of reports have highlighted the substantial morbidity and mortality associated with *M. odoratimimus* infections. Clinical manifestations include septic shock, pneumonia, cellulitis, urinary tract infections (UTIs), recurrent heel ulcer infections, soft tissue infections, and necrotizing fasciitis. These infections are observed most frequently in intensive care unit (ICU) settings, where prolonged urinary catheterization and a high prevalence of immunosuppressed patients create favorable conditions for opportunistic pathogens.

Currently, research on the antibiotic resistance of *M. odoratimimus* is limited. Available studies have largely focused on its resistance to conventional antibiotics, particularly β-lactam antibiotics (Venuti et al., 2023). Accumulating evidence indicates that M. odoratimimus exhibits high-level resistance to multiple commonly used antibiotics, including penicillins, cephalosporins, aminoglycosides, aztreonam, and carbapenems (Tran and Juergens, 2023; Ogiso-Tanaka et al., 2022). Genomic sequencing and bioinformatics analysis have revealed a range of virulence factors and various antibiotic-resistance genes in *M. odoratimimus* (Aborode et al., 2022; Khojasteh-Leylakoohi et al., 2023). Among these, the metallo-β-lactamase gene (*bla*_MUS-1_), which confers intrinsic resistance to β-lactam antibiotics, has been predicted to be present in all analyzed *M. odoratimimus* strains. Managing *M. odoratimimus* infections poses a significant challenge due to its multidrug resistance, biofilm formation capability, and polysaccharide capsule.

Antibiotic resistance has emerged as a major global public health threat (Jean et al., 2022; Hoenigl et al., 2021; Arastehfar et al., 2021). The management of infections in immunocompromised individuals, particularly patients with cancer, is especially challenging (Aliabadi et al., 2022; Jiang et al., 2023; Copaescu et al., 2023). Given the multidrug-resistant profile of *M. odoratimimus*, therapeutic options remain limited and clinical outcomes are frequently suboptimal. Emerging evidence indicates that urinary pH can significantly influence antibiotic activity, thereby affecting treatment efficacy (Ali et al., 2023; Plummer et al., 2021). Investigating the antibiotic susceptibility of *M. odoratimimus* under different pH conditions may improve understanding of its resistance behavior and inform optimized antimicrobial strategies.

Despite increasing recognition of *M. odoratimimus* as an opportunistic pathogen in immunocompromised hosts, its resistance mechanisms and infection characteristics in cancer patients remain poorly defined. In particular, UTIs caused by *M. odoratimimus* are rarely reported, and their clinical management is further complicated by multidrug resistance and environmental factors influencing antibiotic performance. A multidrug-resistant *M. odoratimimus* strain isolated from the urine of a gastric cancer patient was characterized, and the impact of environmental pH on antibiotic susceptibility was systematically investigated both *in vitro* and *in vivo*. These findings provide novel insights into the management of multidrug-resistant *Myroides* infections and broaden current understanding of this emerging pathogen in oncology settings.

## 2 Materials and methods

### 2.1 Patients and isolates

A strain of *M. odoratimimus* was isolated from the urine of a 61-year-old patientwith gastric cancer at West China School of Public Health and West China Fourth Hospital, Sichuan University (Chengdu, China). Clinical data were collected, including comorbidities, indwelling catheterization, antimicrobial therapy, and clinical outcomes. Urine samples were cultured on MacConkey agar at 37 °C for 48 hours, yielding pale yellow colonies with uniform morphology. Colony purity was verified by Gram staining and three successive subcultures on blood agar plates. Pure colonies were suspended in sterile phosphate-buffered saline (PBS, pH 7.6) and adjusted to a turbidity equivalent to a 0.5 McFarland standard for subsequent experiments (**Supplementary Figure 1**).

### 2.2 Identification of isolates

Microbial identification and antimicrobial susceptibility testing (AST) were performed using the VITEK Compact 15 system and VITEK MS system (bioMérieux, France) in accordance with the National Clinical Laboratory Procedures Guidelines.

16S ribosomal ribonucleic acid (16S rRNA) sequencing was conducted to identify the bacterial species. The genomic DNA of the isolated bacteria was extracted and amplified using universal primers 27F (5’-AGTTTGATCCTGGCTCAG-3’) and 1492R (5’-GGTTACCTTGTTACGACTT-3’). The 16S rRNA gene sequence was then compared with data in the NCBI GenBank database, confirming the isolate as *M. odoratimimus* PR63069.

### 2.3 AST

The minimum inhibitory concentrations (MICs) of ampicillin, amoxicillin/clavulanic acid, amikacin, aztreonam, ciprofloxacin, colistin, ceftriaxone, cefazolin, doxycycline, ceftazidime, nitrofurantoin, gentamicin, imipenem, levofloxacin, meropenem, minocycline, piperacillin, cefoperazone/sulbactam, trimethoprim-sulfamethoxazole, cefotaxime, tetracycline, tigecycline, tobramycin, and piperacillin/tazobactam were determined using the VITEK 2-Compact 15 system with the Gram-negative susceptibility card (AST-GN14) (bioMérieux, France). The assay was conducted once in accordance with routine clinical microbiology laboratory procedures. Results were interpreted according to the Clinical and Laboratory Standards Institute (CLSI) criteria for non-Enterobacterales organisms, as previously described (Tian et al., 2016).

### 2.4 Detection of antibiotic resistance genes

The genomic DNA of *M. odoratimimus* was extracted through thermal lysis. The primers used to detect 13 antibiotic resistance genes and the corresponding PCR conditions are listed in **Table 1** and **Table 2**, respectively. The PCR reaction steps were as follows: initial denaturation at 95 °C for 5 minutes, followed by 35 cycles of denaturation at 94 °C for 40 seconds, annealing at 52-63 °C for 30 seconds, and extension at 72 °C for 1 minute, with a final extension at 72 °C for 10 minutes. Amplified products were analyzed by agarose gel electrophoresis in three independent experiments. Positive amplicons were purified and sequenced by Tsingke (Beijing, China). Sequence alignment and analysis were conducted using DNAMAN 6.0 software, and homology searches were performed using the NCBI BLAST database.

**Table 1:** The primer sequences for antibiotic resistance gene screening.

Gene	Primer	Sequence (5'-3')
*bla*_MUS-1_/ *bla*_MUS-2_/ *bla*_MUS-3_	MUS-1-F	5'-GGTCATCACTACCCACTTCCAC-3'
	MUS-1-R	5'-AAGCTATCACGTTACCATCGGC-3'
*bla*_IMP-1_	IMP-1-F	5'-CTACCGCAGCAGAGTCTTTG-3'
	IMP-1-R	5'-AACCAGTTTTGCCTTACCAT-3'
*bla*_VIM-1_	VIM-1-F	5'-AGTGGTGAGTATCCGACAG-3'
	VIM-1-R	5'-ATGAAAGTGCGTGGAGAC-3'
*bla*_NDM-1_	NDM-1-F	5'-CTTCCAACGGTTTGATCGTC-3'
	NDM-1-R	5'-ATTGGCATAAGTCGCAATCC-3'
*bla*_OXA-48_	OXA-48-F	5'-TTGGTGGCATCGATTATCGG-3'
	OXA-48-R	5'-GAGCACTTCTTTTGTGATGGC-3'
*bla*_GES_	GES-F	5'-CTTCATTCACGCACTATTAC-3'
	GES-R	5'-TAACTTGACCGACAGAGG-3'
SUL1	SUL1-F	5'-CACCGGAAACATCGCTGCA-3'
	SUL1-R	5'-AAGTTCCGCCGCAAGGCT-3'
SUL2	SUL2-F	5'-CTCCGATGGAGGCCGGTAT-3'
	SUL2-R	5'-GGGAATGCCATCTGCCTTGA-3'
SUL3	SUL3-F	5'-TCCGTTCAGCGAATTGGTGCAG-3'
	SUL3-R	5'-TTCGTTCACGCCTTACACCAGC-3'
ermB	ermB-F	5'-CATGCGTCTGACATCTATCTGA-3'
	ermB-R	5'-CTGTGGTATGGCGGGTAAGTT-3'
qnrA	qnrA-F	5'-AGGATTTCTCACGCCAGGATT-3'
	qnrA-R	5'-CCGCTTTCAATGAAACTGCAA-3'
qnrB	qnrB-F	5'-CAGATTTYCGCGGCGCAAG-3'
	qnrB-R	5'-TTCCCACAGCTCACACTTTTC-3'
qnrS	qnrS-F	5'-GTATAGAGTTCCGTGCGTGTGA-3'
	qnrS-R	5'-GGTTCGTTCCTATCCAGCGATT-3'

**Table 2:** The chemicals and quantity needed for the amplification of antibiotic resistant genes.

Chemicals	Quantity
Bacterial DNA	2.0μL
Forward primer	1.0μL
Reverse primer	1.0μL
2 x PCR Mix	0.5μL
Taq polymerase	0.5μL
ddH_2_O	0.8μL
Total volume	25μL

### 2.5 Effect of pH on antibiotic sensitivity

According to CLSI guidelines, antimicrobial activity was evaluated using the Kirby–Bauer disk diffusion (K–B) method on Mueller–Hinton agar (MHA) plates. A 0.5 McFarland bacterial suspension was evenly spread onto MHA plates using sterile cotton swabs. For single-agent testing, antibiotic disks were placed directly onto the agar surface. For combination assays, minocycline disks served as anchor disks, onto which 10 μL of tigecycline, piperacillin, or tazobactam stock solution was aseptically applied (final amounts per disk: 15 μg tigecycline, 100 μg piperacillin, and 10 μg tazobactam). Disks were air-dried at room temperature for 10–15 min before placement on the inoculated plates. The tested combinations included minocycline + tigecycline, minocycline + piperacillin, and minocycline + tazobactam. Plates were incubated at 37 °C with 5% CO_2_ for 18 ± 2 h, and inhibition zone diameters were measured in millimeters. All experiments were performed in triplicate.

### 2.6 *In vivo* experiment validation

An *in vivo* UTI model was established to evaluate the effect of urine pH on the therapeutic efficacy of minocycline against *M. odoratimimus*. The model was designed to simulate multidrug-resistant UTI under altered urinary pH conditions in immunocompromised hosts.

Fifty male C57BL/6NCrl mice were obtained from Beijing Vital River Laboratory Animal Technology Co., Ltd. Mice were randomly assigned to four groups: control group (n = 10), uninfected and without pH adjustment; infection group (n = 20), infected with *M. odoratimimus* without pH modulation; low-pH group (n = 10), infected and treated with ammonium chloride to acidify urine; and high-pH group (n = 10), infected and treated with sodium bicarbonate to alkalinize urine. To modulate urinary pH, 0.5% ammonium chloride or sodium bicarbonate was added to the drinking water of the respective groups starting one day prior to infection and maintained throughout the experiment. A bacterial suspension containing 1 × 10^8^ CFU of *M. odoratimimus* was inoculated intravesically to establish infection. Twenty-four hours post-infection, minocycline was administered intraperitoneally to all infected groups at 10 mg/kg once daily for five consecutive days. Urine pH was monitored daily using pH test strips (Beyotime Biotechnology). After completion of treatment, bacterial loads were quantified. Body weight, food intake, activity level, and signs of distress were recorded throughout the study. All animal procedures were conducted in accordance with national guidelines for the care and use of laboratory animals and were approved by the Institutional Animal Ethics Committee (Production License No. SCXK (Chuan) 2020-003). Animals were housed in barrier facilities, and efforts were made to minimize suffering and reduce animal use.

### 2.7 Combination drug study

The *in vitro* synergistic effects of antibiotic combinations were evaluated using the checkerboard microdilution method. Clinical isolates of *M. odoratimimus* were cultured in Mueller–Hinton broth (MHB) and tested according to CLSI standards. The following combinations were assessed: minocycline + tigecycline, minocycline + piperacillin, and minocycline + tazobactam (Sigma-Aldrich).

Bacterial cultures were grown at 37 °C to the logarithmic phase and adjusted to a 0.5 McFarland standard (approximately 1.5 × 10^8^ CFU/mL) using a nephelometer. The suspension was further diluted in sterile saline to achieve a final inoculum of 1 × 10^6^ CFU/mL, which was confirmed by plate counting. Antibiotic stock solutions were prepared in MHB and serially diluted. Minocycline was tested at concentrations ranging from 0.125 to 32 μg/mL, while tigecycline, piperacillin, and tazobactam were tested at 0.5 to 128 μg/mL. Drug combinations were arranged in a two-dimensional checkerboard format in 96-well microplates. Each well contained a total volume of 300 μL, resulting in a final bacterial inoculum of 5 × 10^5^ CFU/mL. Plates were incubated at 37 °C for 24 hours. Bacterial growth was determined by measuring optical density at 600 nm (OD_600_) using a microplate reader (Multiskan FC, Thermo Fisher Scientific). The fractional inhibitory concentration (FIC) index was calculated for each combination as the sum of the FICs of the two agents. Synergy was defined as FIC index < 0.5, additivity as 0.5 ≤ FIC index ≤ 4, and antagonism as FIC index > 4. Data were expressed as mean ± standard deviation (SD). Statistical analysis was performed using SPSS 26.0, and *p* < 0.05 was considered statistically significant. Synergy plots were generated using GraphPad Prism 9.0.

### 2.8 Host cell-bacteria interactions

Fifty immunodeficient NOD.Cg-PrkdcscidIl2rgtm1Sug/JicCrl mice (20–25 g; Beijing Vital River Laboratory Animal Technology Co., Ltd.) were randomly assigned to five groups (n=10 per group): control, infection (1×10^7^ CFU), low-dose infection (1×10^6^ CFU), high-dose infection (1×10^8^ CFU), and treatment group. The control group received no intervention. Systemic infection was induced by tail vein injection of *M. odoratimimus* at the indicated inocula. In the treatment group, minocycline (10 mg/kg; Sigma-Aldrich) was administered intraperitoneally once daily for five consecutive days, beginning within 24 hours after infection with the standard inoculum. Mice were maintained under specific pathogen-free conditions with a 12-h light/dark cycle. At the end of the treatment period, blood samples were collected for bacterial quantification by agar plate counting. Serum concentrations of tumor necrosis factor-α (TNF-α), interleukin-6 (IL-6), and interferon-γ (IFN-γ) were measured using enzyme-linked immunosorbent assay (ELISA) kits (Beyotime Biotechnology).

### 2.9 Statistical analysis

All data analyses and graphing were performed using GraphPad Prism 9.0 software. Continuous variables are presented as mean ± standard deviation (SD). Group comparisons were conducted using ANOVA followed by Tukey’s multiple comparison test. Synergistic effects were evaluated based on the fractional inhibitory concentration (FIC) index, where applicable. A two-sided *p-*value < 0.05 was considered statistically significant.

## 3 Results

### 3.1 Patient case

A 61-year-old male with a 7-year history of gastric malignancy was admitted on March 24, 2020, to the Department of Palliative Medicine at West China School of Public Health and West China Fourth Hospital, Sichuan University, due to severe gastric pain and bleeding. He had previously undergone subtotal gastrectomy followed by eight cycles of postoperative chemotherapy, with initial satisfactory recovery.

Two months prior to admission, progressive back pain developed, followed by acute bilateral lower limb weakness one month later. Evaluation at a local hospital revealed spinal metastases with spinal cord compression syndrome, epilepsy, gastrointestinal dysfunction, severe anemia, and urinary tract infection (UTI) with hematuria. Lumbar radiotherapy was initiated but discontinued because of poor tolerance. Conservative management included levofloxacin for recurrent UTI and analgesics for cancer-related pain; however, hematuria and low-grade fever persisted. Escalating pain required fentanyl transdermal patches, with limited relief. The patient was subsequently transferred for palliative management. On admission, diagnoses included advanced gastric carcinoma with bone metastases, hepatic failure, electrolyte disturbances, and cancer-related pain. Comprehensive supportive treatment was initiated. Three days later, chills and high-grade fever (38.4–39.0 °C) developed, accompanied by dark brown hematuria. Blood and urine cultures were obtained due to clinical deterioration.

Urine culture identified *M. odoratimimus*. Based on AST, minocycline therapy was initiated, resulting in resolution of fever. Despite temporary infection control, the patient developed progressive jaundice and severe scleral icterus. Percutaneous transhepatic biliary drainage was recommended but declined by the family. Palliative sedation was initiated for refractory pain. The patient died on April 2, 2020. The terminal event was attributed to multi-organ failure in the context of advanced malignancy, hepatic failure, and persistent multidrug-resistant urinary tract infection.

### 3.2 The isolated strain was resistant to all 24 tested antibiotics, except minocycline

**Figure 1:**
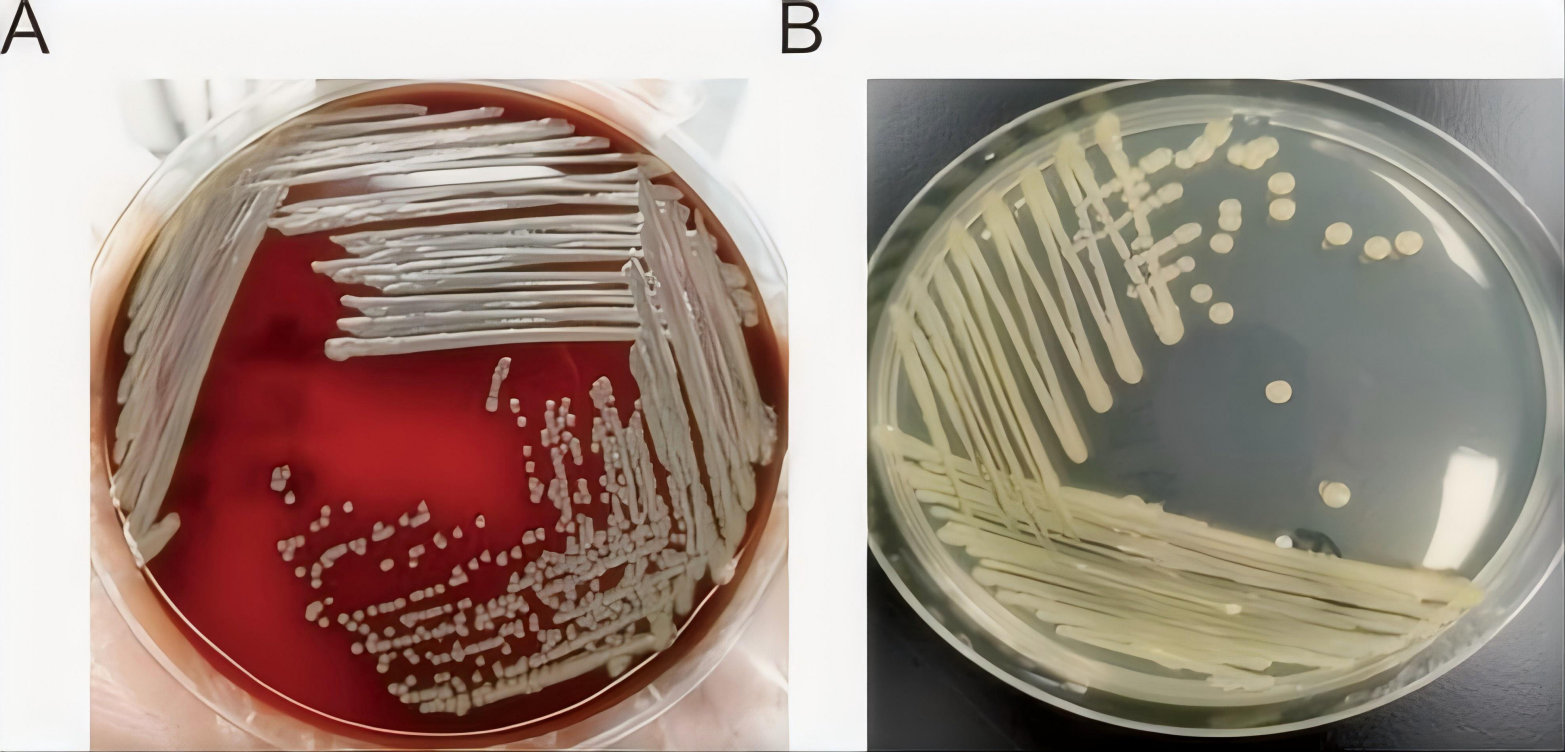
Colony morphology of *M. odoratimimus*. **(A)** Blood agar plate: Round, smooth, pale yellow colonies after 24 hours of incubation at 37°C. **(B)** MacConkey agar plate: Moist, glossy, pale pink colonies indicating non-lactose fermentation.

After 24 hours of incubation at 37 °C, a single colony was isolated from the urine sample. The colony appeared circular, smooth, yellow-pigmented, and emitted a characteristic foul odor (**Figure 1**). Initial identification using VITEK2-compact15 based on conventional biochemical reactions suggested Myroides species but did not allow precise species-level discrimination. Matrix-assisted laser desorption/ionization time-of-flight mass spectrometry (VITEK MS) subsequently identified the isolate as *M. odoratimimus* (matching score > 2.2, sequence homology > 99%). Sequencing of the approximately 1,500-bp *16S rRNA* gene demonstrated 100% identity with *M. odoratimimus* PR63069 in the GenBank database, confirming species-level identification.

AST revealed an extensive drug-resistant phenotype (**Table 3**). The isolate exhibited resistance to 24 antibiotics across seven major classes, including aminoglycosides, β-lactams, polypeptides, quinolones, tetracyclines, nitrofurans, and sulfonamides. Notably, susceptibility was retained only to minocycline.

**Table 3:** Antibiotic susceptibility test result of M. odoratimimus.

Antibiotic			MIC (µg/mL)	Susceptibility
Aminoglycosides		Amikacin	≥64	R
		Gentamicin	≥16	R
		Tobramycin	≥16	R
β-lactams	Penicillins	Ampicillin	≥32	R
		Amoxicillin/Clavulanate	≥32	R
		Piperacillin	≥128	R
		Piperacillin/Tazobactam	≥128	R
	Carbapenems	Imipenem	≥16	R
		Meropenem	≥16	R
	Monobactams	Aztreonam	≥64	R
	Cephalosporins	Cefazolin	≥64	R
		Ceftazidime	≥64	R
		Ceftriaxone	≥64	R
		Ceftizoxime	≥32	R
		Cefoperazone/Sulbactam	≥64	R
Polypeptides		Colistin	≥16	R
Quinolones		Ciprofloxacin	≥4	R
		Levofloxacin	≥8	R
Tetracyclines		Tetracycline	≥16	R
		Minocycline	2	S
		Tigecycline	≥8	R
		Doxycycline	≥16	R
Nitrofuran		Nitrofurantoin	256	R
Sulfonamides		Trimethoprim/Sulfamethoxazole	≥320	R

MIC, minimum inhibitory concentration.

### 3.3 Detection of multiple resistance genes and specific amplification of the *bla*_MUS-1_ gene in the *M. odoratimimus* strain

**Figure 2:**
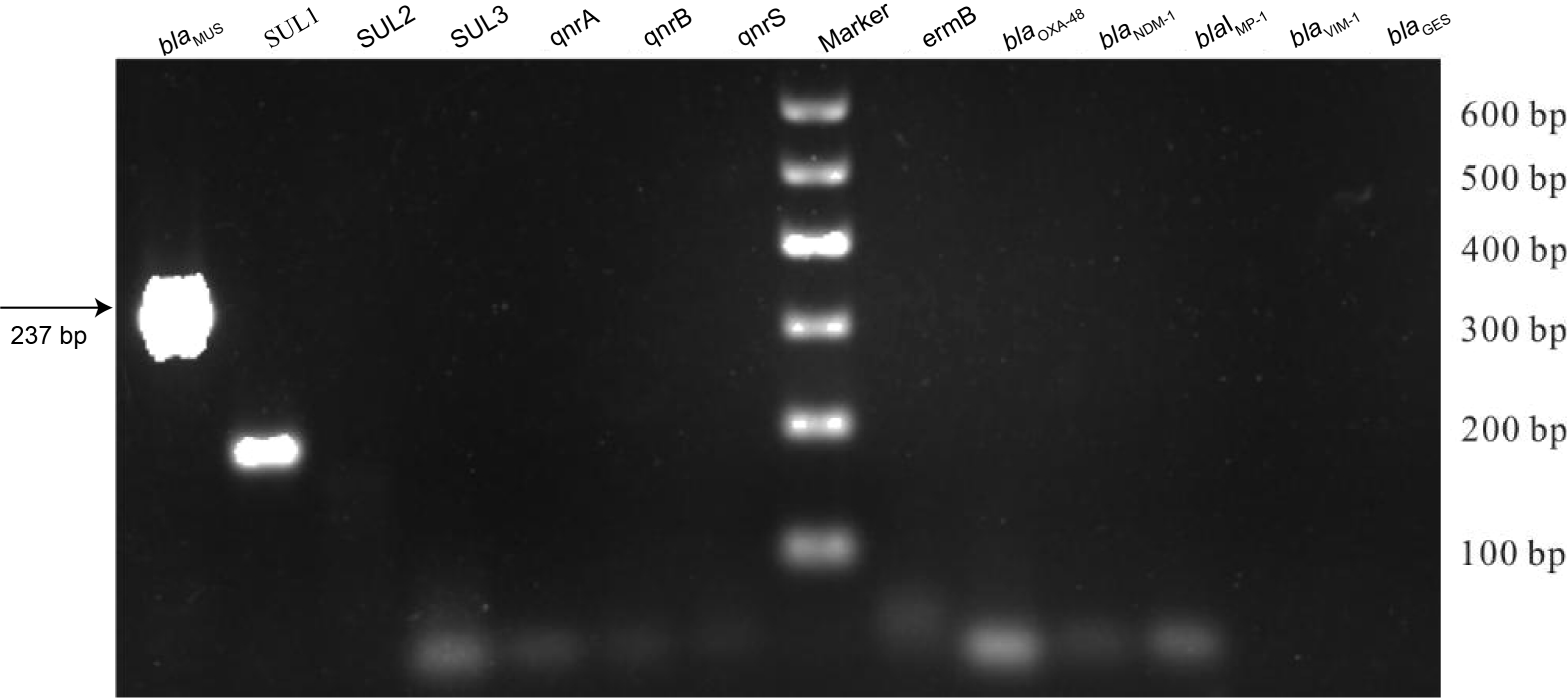
PCR detection of antibiotic resistance genes in clinically isolated *M. odoratimimus* strains. PCR products of tested antibiotic resistance genes are shown. Marker: DNA ladder. The label above each lane indicates the target gene for each amplification system. The experiment was performed in triplicate (three independent repeats), and representative results were presented.

Thirteen resistance genes were screened, including carbapenemase-encoding genes (*bla*_MUS-1_, *bla*_IMP-1_, *bla*_NDM-1_, *bla*_VIM-1_, *bla*_OXA-48_, and *bla*des) (Watanabe et al., 1991; Smith et al., 2007; Cuzon et al., 2008; Dallenne et al., 2010; Poirel et al., 2011; Al-Bayssari et al., 2015, F. Mojica et al., 2016); sulfonamide resistance genes (*sul1*, *sul2*, *sul3*) (Sundström et al., 1988; Perreten and Boerlin, 2003; Byrne-Bailey et al., 2009); macrolide resistance gene (*ermB*) (Gevers et al., 2003); and quinolone resistance genes (*qnrA*, *qnrB*, *qnrS*) (Mendes et al., 2008; Carattoli et al., 2010; Michon et al., 2011). PCR was performed using appropriate primers for further screening (**Table 4**). Two bands related to MUS-1 and SUL1 were amplified, recovered, and sequenced (**Figure 2**). However, nucleotide sequence analysis revealed that only the chromosomal MUS-1 gene was amplified in this strain, primarily due to non-specific amplification using SUL1 primers.

**Table 4:** Inhibition zones (mm) of different antibiotic measured by K-B method.

Antibiotics	pH					
	5.5	6.5	7.3	7.9	8.4	9.0
Blank	0	0	0	0	0	0
Amikacin	0	0	0	0	0	0
Piperacillin/tazobactam	0	0	6.0 ± 0.8	9.0 ± 1.1	12.0 ± 1.2	9.0 ± 0.7
Ceftazidime/avibactam	0	0	0	0	0	0
Cefoperazone/sulbactam	0	0	0	0	0	0
Gentamicin	0	0	0	0	0	0
Tigecycline	0	0	14.0 ± 0.5	12.5 ± 1.1	0	8.0 ± 1.0
Minocycline	0	0	21.0 ± 1.1	19.0 ± 1.6	7.0 ± 1.0	0

### 3.4 Tigecycline and minocycline exhibit better antibacterial activity at neutral to slightly alkaline pH values (7.3 to 9.0)

To evaluate the influence of environmental pH on antibiotic efficacy, *M. odoratimimus* was cultured on MHA plates adjusted to pH values of 5.5 ± 0.1, 6.5 ± 0.1, 7.31 ± 0.1, 7.9 ± 0.1, 8.4 ± 0.1, and 9.0 ± 0.1. Antibiotic susceptibility testing was performed under each pH condition.

**Figure 3:**
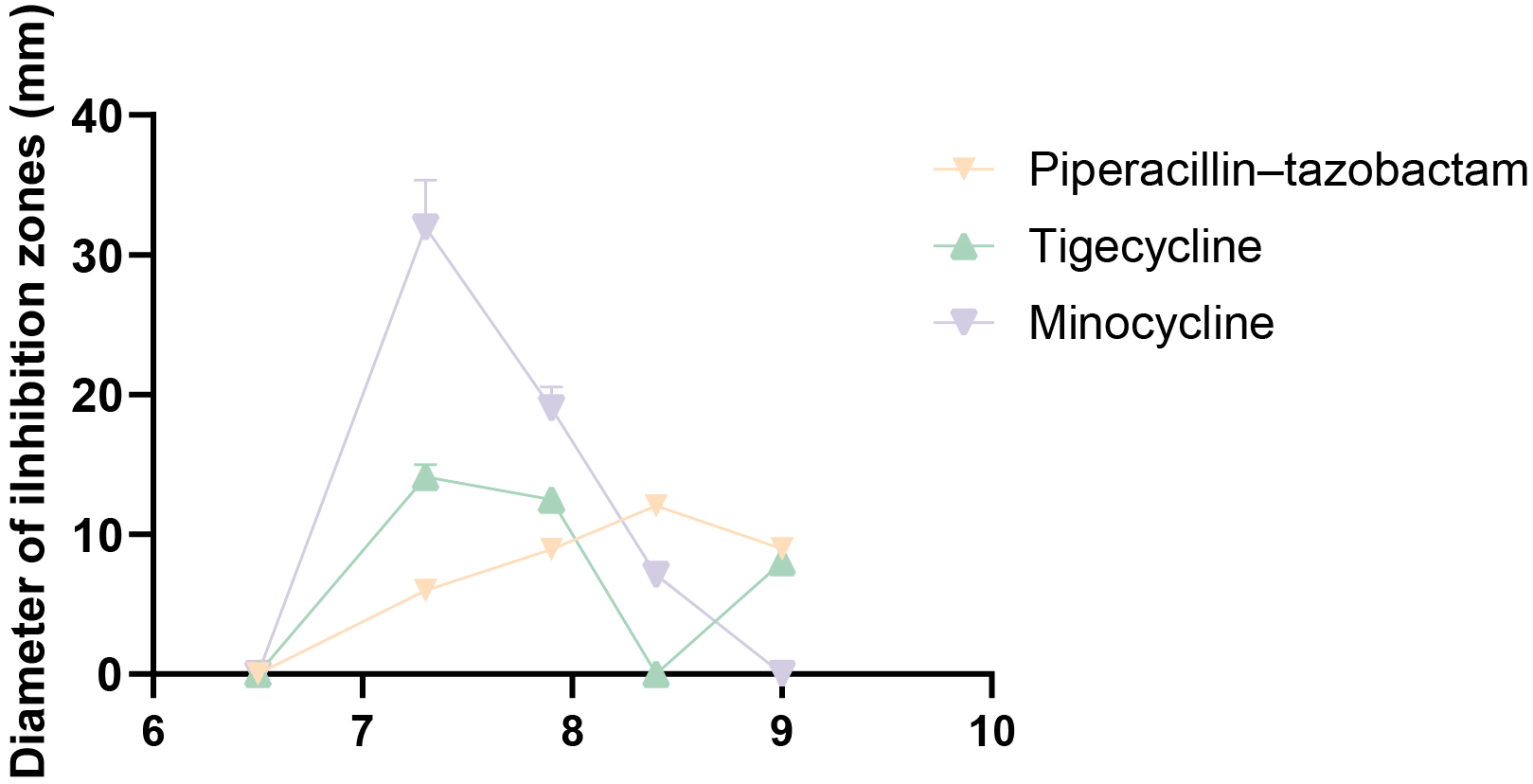
Inhibitory effects of three antibiotics against *M. odoratimimus* under different pH conditions. The inhibitory activities of three antibiotics at different pH values (5.5–9.0) were determined using the disk diffusion method. Piperacillin-tazobactam (yellow); tigecycline (green); minocycline (purple). The experiment was performed in triplicate (three independent repeats).

No inhibition zones were observed for amikacin, ceftazidime/avibactam, cefoperazone/sulbactam, or gentamicin at any tested pH level. In addition, none of the tested antibiotics exhibited measurable activity under acidic conditions (pH 5.5 and 6.5) (**Table 4**). In contrast, piperacillin–tazobactam, tigecycline, and minocycline displayed pH-dependent antibacterial activity at neutral to alkaline conditions (pH 7.3–9.0) (**Figure 3**). Tigecycline and minocycline, both tetracycline-class agents, showed comparable antibacterial spectra, with the largest inhibition zones observed at pH 7.3 (14 ± 0.5 mm and 21 ± 1.1 mm, respectively). Piperacillin–tazobactam exhibited maximal activity at pH 8.4, with an inhibition zone of 12.0 ± 1.2 mm.

### 3.5 Study on a mouse infection model based on the impact of urine pH regulation on minocycline’s antibacterial efficacy

**Figure 4:**
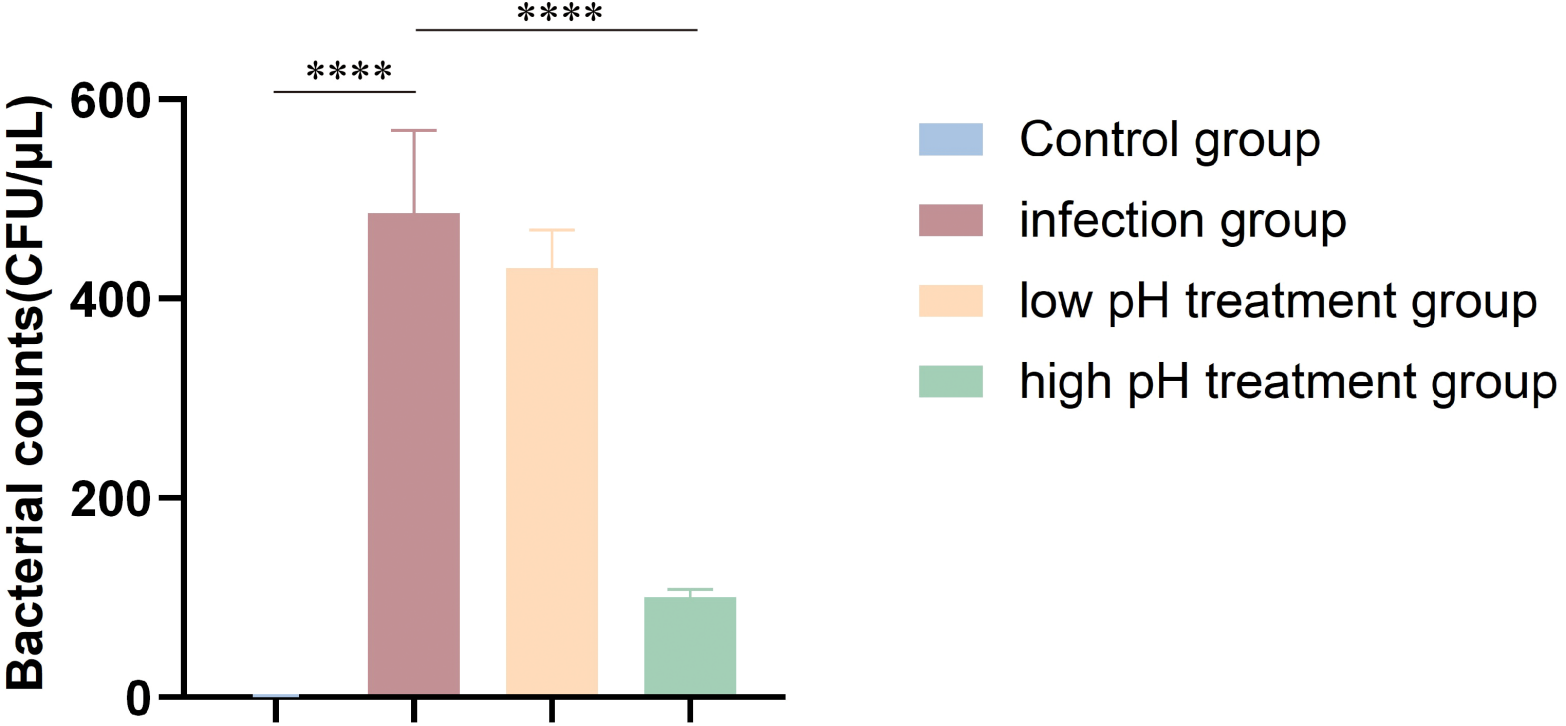
Effect of urine pH on the *in vivo* antibacterial activity of minocycline. Comparison of bacterial load (log_10_ CFU/μL) in bladder tissues among different groups. ****p < 0.0001. The infection group was inoculated with 1 × 10^8^ colony-forming units (CFU) per mouse, with n = 10 per group.

Urinary pH regulation in mice was stable throughout the experiment, with mean values of approximately 7.0 in the control group, 5.5 in the low-pH group, and 8.5 in the high-pH group. Bladder bacterial loads were significantly elevated in the infection group compared with the control group, confirming successful establishment of the infection model. Following minocycline treatment, the high-pH group demonstrated a significantly greater reduction in bacterial burden than both the untreated infection group and the low-pH group (**Figure 4**), indicating enhanced antibacterial efficacy under alkaline conditions. In contrast, bacterial clearance was markedly attenuated in the low-pH group. No significant differences in body weight were observed among groups before the experiment (**Supplementary Figure 2A**). After treatment, mice in the high-pH group exhibited the least reduction in body weight and activity levels, whereas the low-pH group showed the most pronounced weight loss and decreased activity (**Supplementary Figure 2B**). These results suggest that urine pH can influence the therapeutic effect of minocycline against *M. odoratimimus* infection.

### 3.6 Combination of minocycline and tigecycline exhibits superior synergistic antibacterial activity

**Figure 5:**
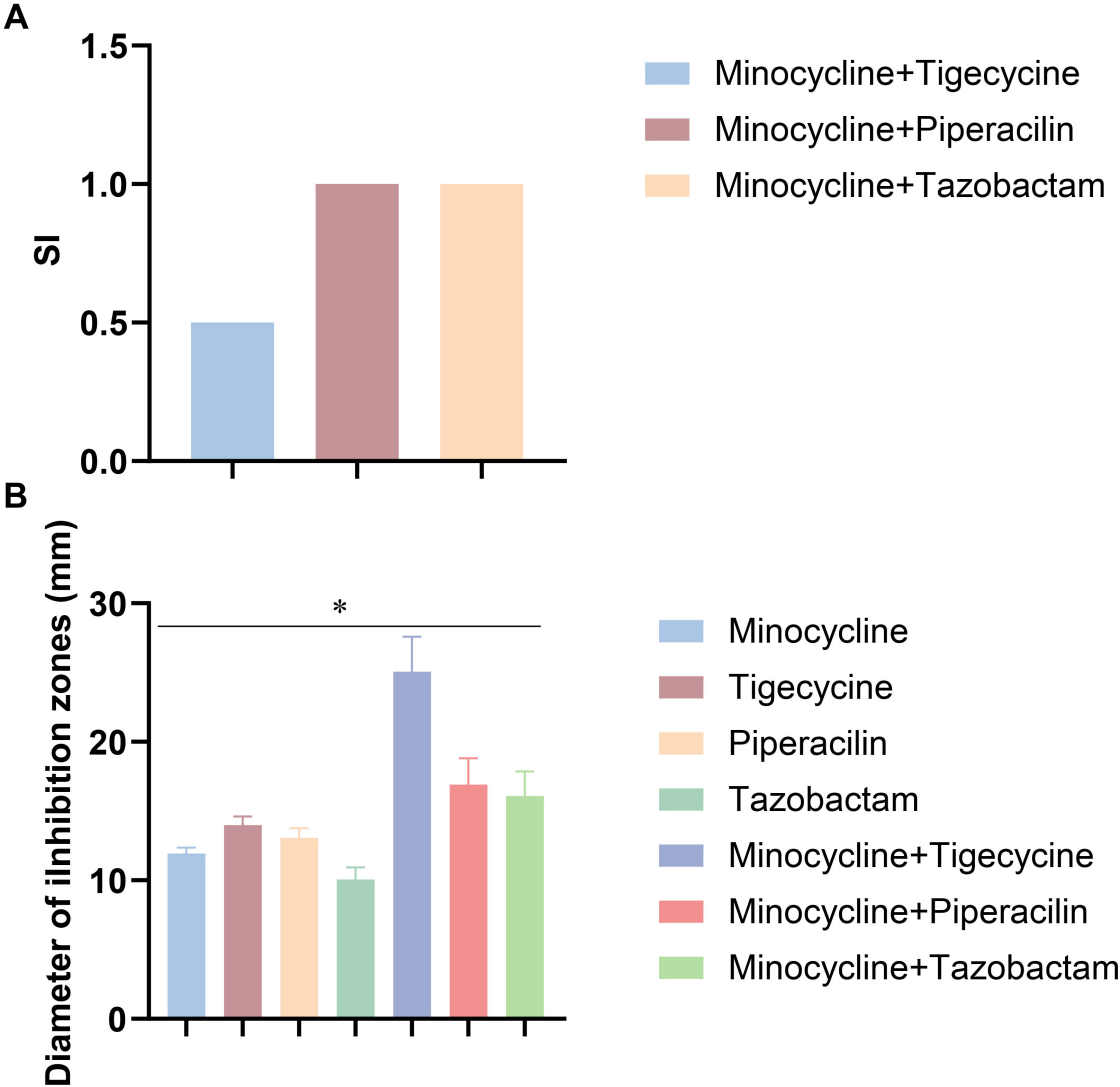
Synergistic antibacterial effect of minocycline combined with tigecycline. **(A)** Fractional inhibitory concentration (FIC) index of antibiotic combinations determined by the checkerboard assay. FIC index < 0.5 indicates synergy; 0.5–4 indicates additivity; > 4 indicates antagonism. **(B)** Inhibition zone diameters of antibiotic monotherapy and combinations against Myroides odoratimimus. Data are presented as mean ± SD from three independent experiments. *p < 0.05 between indicated groups.

Checkerboard analysis revealed a synergistic interaction between minocycline and tigecycline against *M. odoratimimus* (**Figure 5A**). The FIC index was < 0.5, indicating synergy. In contrast, the combination of minocycline and piperacillin/tazobactam yielded an FIC index of 1.0, consistent with an additive effect without synergy or antagonism. These results suggest that the minocycline–tigecycline combination provides superior synergistic antibacterial activity (**Figure 5B**) and may represent a promising therapeutic strategy for multidrug-resistant *M. odoratimimus* infections.

### 3.7 *M. odoratimimus* infection significantly enhances the host immune response and is affected by antibiotic treatment

**Figure 6:**
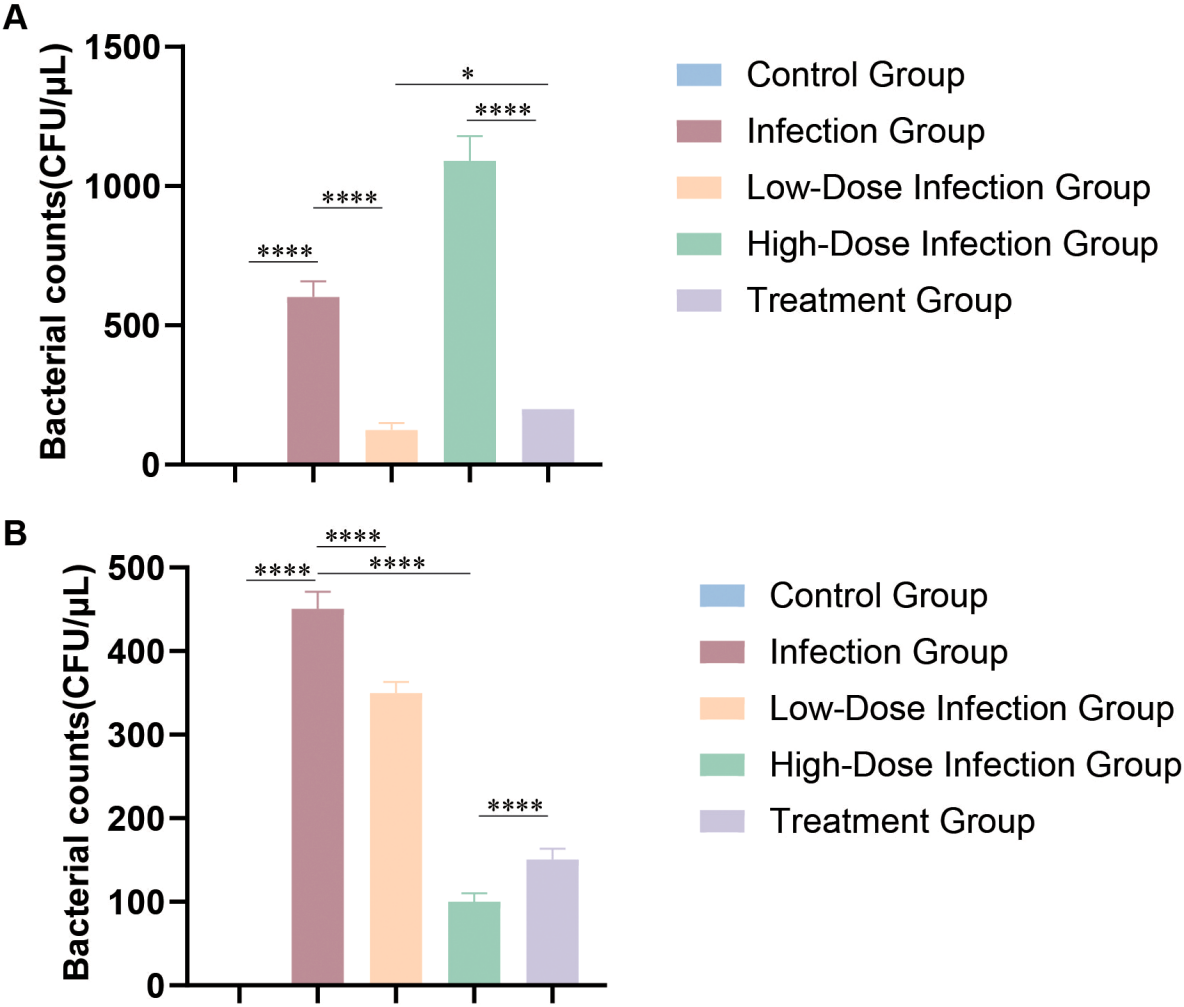
Clearance of *M. odoratimimus* under different treatment regimens. **(A)** Bacterial load of *M. odoratimimus* in the blood of mice from each infected group (standard dose, low dose, high dose) at 24 hours post-infection. **(B)** Bacterial load in the treatment group one week post-infection. *p < 0.05, ****p < 0.0001. The infection group was inoculated with 1 × 10^8^ colony-forming units (CFU) per mouse, with n = 10 per group.

**Figure 7:**
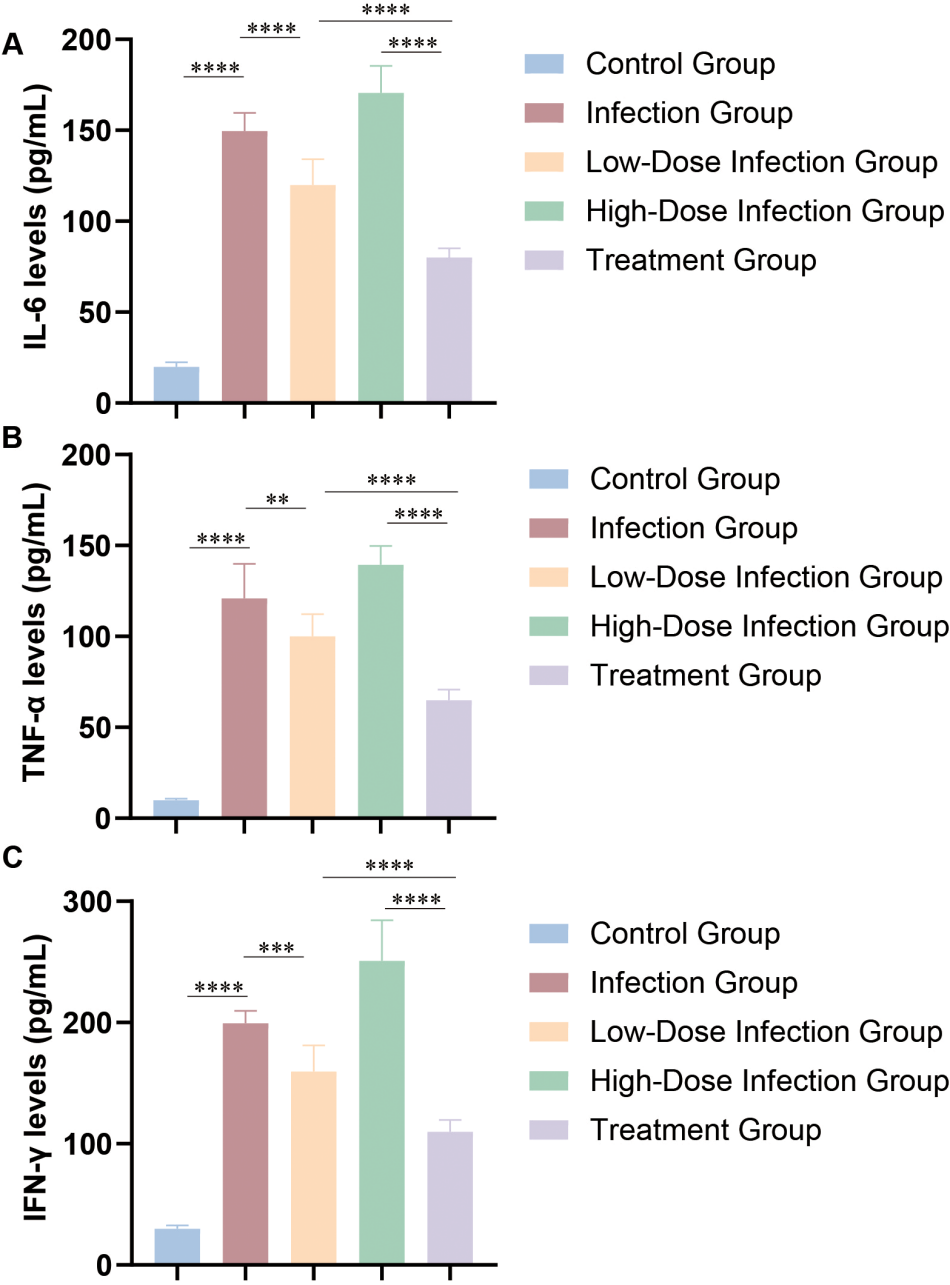
Effect of *M. odoratimimus* infection on host inflammatory cytokine levels. **(A)** Serum IL-6 levels in each group. **(B)** Serum TNF-α levels in each group. **(C)** Serum IFN-γ levels in each group. ** indicates *p* < 0.01; *** indicates *p* < 0.001; **** indicates *p* < 0.0001. The infection group was inoculated with 1 × 10^8^ colony-forming units (CFU) per mouse, with n = 10 per group (three independent repeats).

At 24 hours post-infection, bacterial loads in the blood and spleen were significantly higher in all infected groups (low-, standard-, and high-dose) compared with the control group (*p* < 0.01; **Figure 6A**). The high-dose infection group showed significantly higher bacterial loads than the standard and low-dose groups (*p* < 0.05), indicating rapid bacterial proliferation *in vivo*. At one week post-infection, mice receiving minocycline treatment showed a marked reduction in bacterial load compared with the untreated infection group (*p* < 0.01; **Figure 6B**), demonstrating the *in vivo* antibacterial efficacy of minocycline. Serum levels of TNF-α, IL-6, and IFN-γ in all infected groups were significantly elevated compared to controls (*p* < 0.001). Although cytokine concentrations were significantly reduced in the treatment group compared with the untreated infection group, they remained higher than baseline levels observed in controls (**Figure 7**). Collectively, these findings indicate that *M. odoratimimus* infection triggers a pronounced inflammatory response, and minocycline treatment partially mitigates both bacterial burden and host inflammatory activation without fully restoring immune homeostasis.

## 4 Discussion

The global emergence of multidrug-resistant and pan-drug-resistant pathogens represents a major challenge to contemporary infectious disease management (Corona et al., 2023; Soriano and Mensa, 2022; Dutta et al., 2022). Within this context, *M. odoratimimus* has increasingly been recognized as an opportunistic Gram-negative pathogen affecting immunocompromised hosts. Although environmental in origin, it has been isolated from diverse clinical specimens, particularly in patients with underlying malignancies or invasive procedures (Dhasarathan et al., 2021; Tolani et al., 2020). The present case highlights the clinical relevance of *M. odoratimimus* infection in oncology settings and underscores the need for heightened vigilance.

This study characterized the antimicrobial susceptibility profile of a urinary *M. odoratimimus* isolate from a cancer patient and investigated the impact of environmental pH on antibiotic efficacy. The isolate exhibited extensive drug resistance, remaining susceptible only to minocycline. Molecular analysis confirmed the presence of the blaMUS-1 resistance gene. Antibiotic activity was markedly affected by pH conditions. Tigecycline and minocycline demonstrated optimal antibacterial effects under neutral conditions, whereas piperacillin–tazobactam showed relatively enhanced activity at higher pH levels. *In vivo* experiments further demonstrated that urinary alkalization significantly improved the therapeutic efficacy of minocycline. Collectively, these findings indicate that, in addition to intrinsic genetic resistance determinants, microenvironmental factors such as urinary pH play a critical role in modulating antibiotic effectiveness against *M. odoratimimus*.

Consistent with previous reports (Boyd et al., 2022; Hu et al., 2023; Mehrotra et al., 2023), the isolate exhibited high-level resistance to multiple β-lactam antibiotics, including cephalosporins and carbapenems. Molecular analysis confirmed the presence of the blaMUS-1 resistance gene, which hydrolyzes β-lactam antibiotics and confers resistance to cephalosporins and carbapenems (Al-Bayssari et al., 2015). The detection of blaMUS-1 provides a mechanistic explanation for the observed resistance phenotype in our susceptibility testing results. However, the extensive resistance pattern observed in this isolate suggests that additional mechanisms, such as efflux pumps or permeability alterations, may also contribute to its multidrug-resistant phenotype.

The study also demonstrated that different pH conditions affect the antibacterial activity of tigecycline, minocycline, and piperacillin-tazobactam: tigecycline and minocycline were most effective at pH 7.3, while piperacillin-tazobactam had enhanced activity at higher pH. Previous studies have also shown that environmental pH can alter bacterial susceptibility to certain antibiotics (Obaees et al., 2024; Mel’iantseva et al., 1995), suggesting that pH may play a significant role in the treatment of *M. odoratimimus* infection.

The murine model findings were consistent with the *in vitro* results, indicating that increased urine pH enhanced the therapeutic efficacy of minocycline and providing additional *in vivo* evidence. However, a “high pH without antibiotic treatment” control group was not included, limiting assessment of the independent contribution of pH modulation. Prior studies indicate that urine alkalinization alone exerts only modest effects on bacterial growth (Müller et al., 2022). Future investigations incorporating appropriate control groups are warranted to clarify the relative impact of pH adjustment independent of antimicrobial therapy.

Based on these findings, urinary pH should be considered during the management of *M. odoratimimus* infections, as it may significantly influence antibiotic efficacy. Tigecycline and minocycline demonstrated greater activity under neutral to alkaline conditions, whereas piperacillin–tazobactam showed relatively improved performance at higher pH levels. These observations suggest that urinary pH modulation could serve as a potential adjunct strategy to optimize antimicrobial therapy. The pH-dependent variation in antibiotic activity may be related to altered bacterial membrane permeability and changes in drug ionization status, both of which can influence intracellular drug accumulation (Shen et al., 2020; Indulkar et al., 2025). However, the precise molecular mechanisms underlying these effects warrant further investigation.

The present study provides preliminary evidence linking the multidrug-resistant phenotype of *M. odoratimimus* with environmental pH–dependent susceptibility patterns, thereby supporting individualized therapeutic considerations. Although urinary pH modulation using ammonium chloride or sodium bicarbonate is clinically feasible (Xue et al., 2022), its safety as an adjunct to enhance antibiotic efficacy requires careful evaluation. Dietary ammonium chloride has been reported to induce metabolic acidosis and may exacerbate urinary tract inflammation in experimental models (Purkerson et al., 2022). In addition, sex-related differences in acid–base regulation have been described in mice (Dominguez Rieg et al., 2025), indicating that pH-modulating strategies may require individualized optimization. Given interspecies variability and the complexity of the human urinary microenvironment, translation of these findings into clinical practice should be approached cautiously. Systematic preclinical safety assessments and controlled clinical studies will be necessary to determine the feasibility and safety of urinary pH modulation as an adjunctive therapeutic strategy.

The present study has several limitations. First, the analysis was based on a single clinical isolate with a limited experimental scale, which restricts epidemiological generalizability and may not fully represent the phenotypic and genotypic diversity of *M. odoratimimus*. Second, standardized clinical interpretive breakpoints for the genus *Myroides* are currently unavailable, and the experimental pH conditions do not entirely replicate the complexity of the human urinary microenvironment. These factors necessitate cautious interpretation of the translational implications. In addition, the *in vivo* evaluation focused exclusively on minocycline, and alternative antimicrobial regimens were not systematically assessed. Validation was also not extended to ESKAPE or other clinically prevalent pathogens, and given interspecies variability in resistance mechanisms, the broader applicability of pH-modulating strategies remains uncertain.

Future research should incorporate larger, multicenter collections of clinical isolates to better define resistance patterns and transmission characteristics of *M. odoratimimus*. Mechanistic studies are warranted to elucidate the molecular basis of pH-dependent antibiotic activity, and comprehensive evaluations of combination therapies and emerging antibacterial approaches, such as antimicrobial peptides and nanomedicine-based strategies, should be undertaken against multidrug-resistant strains. With robust preclinical evidence supporting efficacy and safety, carefully designed clinical investigations may then be pursued. Sustained multidisciplinary collaboration will be essential to advance understanding and optimize management of *M. odoratimimus* infections.

## 5 Conclusion

**Figure 8:**
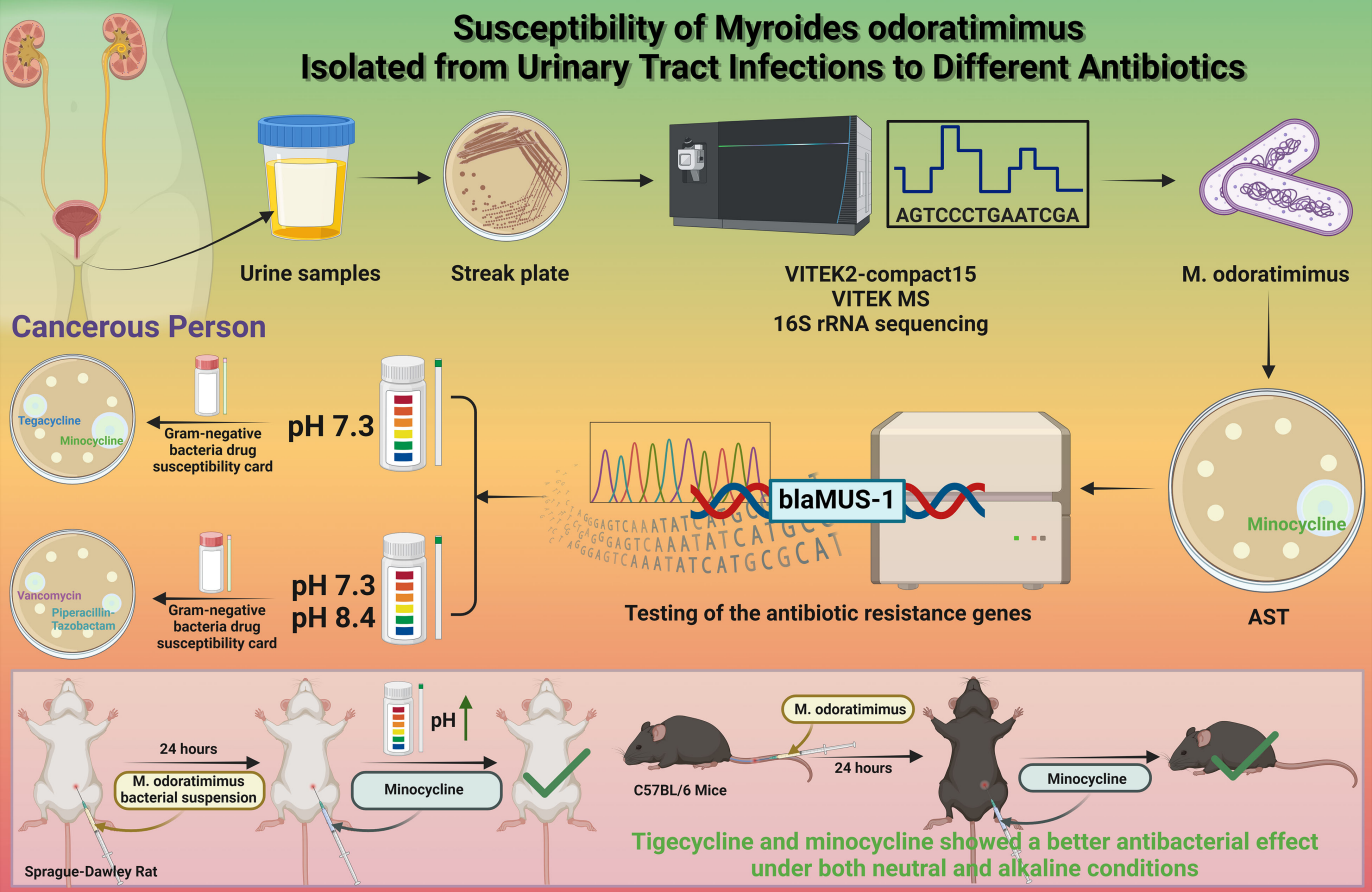
Susceptibility of *M. odoratimimus* isolated from UTIs to different antibiotics.

This study describes a rare case of multidrug-resistant *Myroides odoratimimus* isolated from the urine of a patient with UTI. The isolate exhibited extensive resistance to multiple antibiotics, remaining susceptible only to minocycline. Evaluation under different pH conditions demonstrated that antibiotic activity is significantly influenced by environmental pH, with tigecycline and minocycline showing enhanced efficacy under neutral to alkaline conditions (**Figure 8**). These findings highlight the combined impact of intrinsic resistance mechanisms and microenvironmental modulation on therapeutic outcomes, and provide a potential framework for optimizing treatment strategies against multidrug-resistant *M. odoratimimus* infections.

## Data Availability

The original contributions presented in the study are included in the article/**Supplementary Material**. Further inquiries can be directed to the corresponding author.

## Supplementary material

Supplementary Figure 1 Overview of the experimental procedure for evaluating antibiotic sensitivity of *M. odoratimimus*. This figure illustrates the comprehensive procedure for evaluating the antibiotic sensitivity of *M. odoratimimus* isolated from a UTI. It includes steps from bacterial isolation and identification to antibiotic testing and the impact of pH on antibiotic efficacy, concluding with *in vivo* validation in a mouse model.

Supplementary Figure 2 Body weight changes in mice across different treatment groups. Body weight trends of mice in each group during the experiment. **p* < 0.05, *****p* < 0.0001. The infection group was inoculated with 1 × 10^8^ colony-forming units (CFU) per mouse, with n = 10 per group.
