# Supplementary figures and images for: Unusual case of Myroides odoratimimus infection in a cancer patient: addressing its antimicrobial resistance mechanisms and review of the literature
Source: Front Cell Infect Microbiol. 2026 Apr 30;16:1647777. doi: 10.3389/fcimb.2026.1647777 (PMC13171834; doi:10.3389/fcimb.2026.1647777)

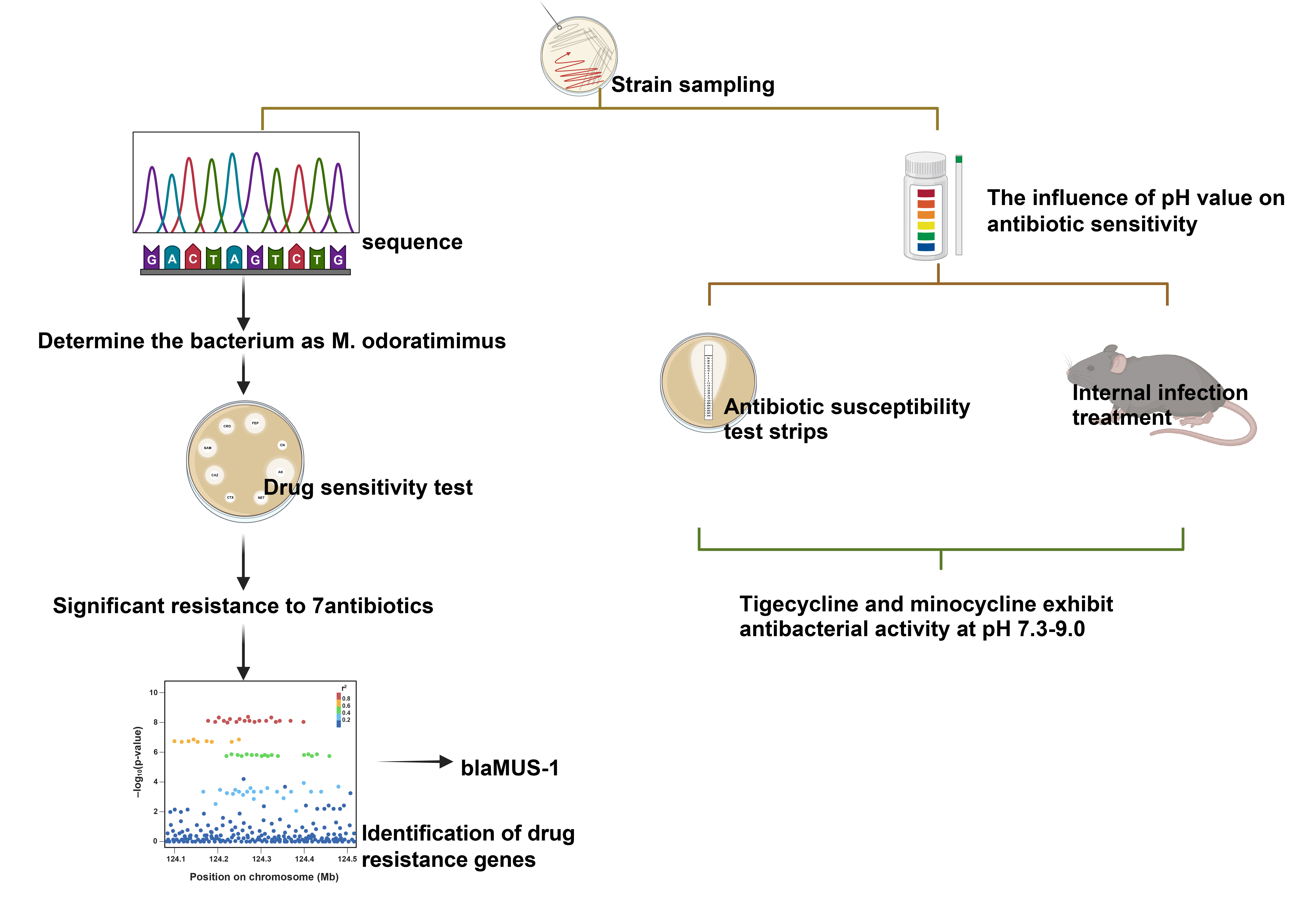

Supplement: Supplementary Figure 1 — Overview of the experimental procedure for evaluating antibiotic sensitivity of M. odoratimimus. This figure illustrates the comprehensive procedure for evaluating the antibiotic sensitivity of M. odoratimimus isolated from a UTI. It includes steps from bacterial isolation and identification to antibiotic testing and the impact of pH on antibiotic efficacy, concluding with in vivo validation in a mouse model. [file Image1.jpeg]

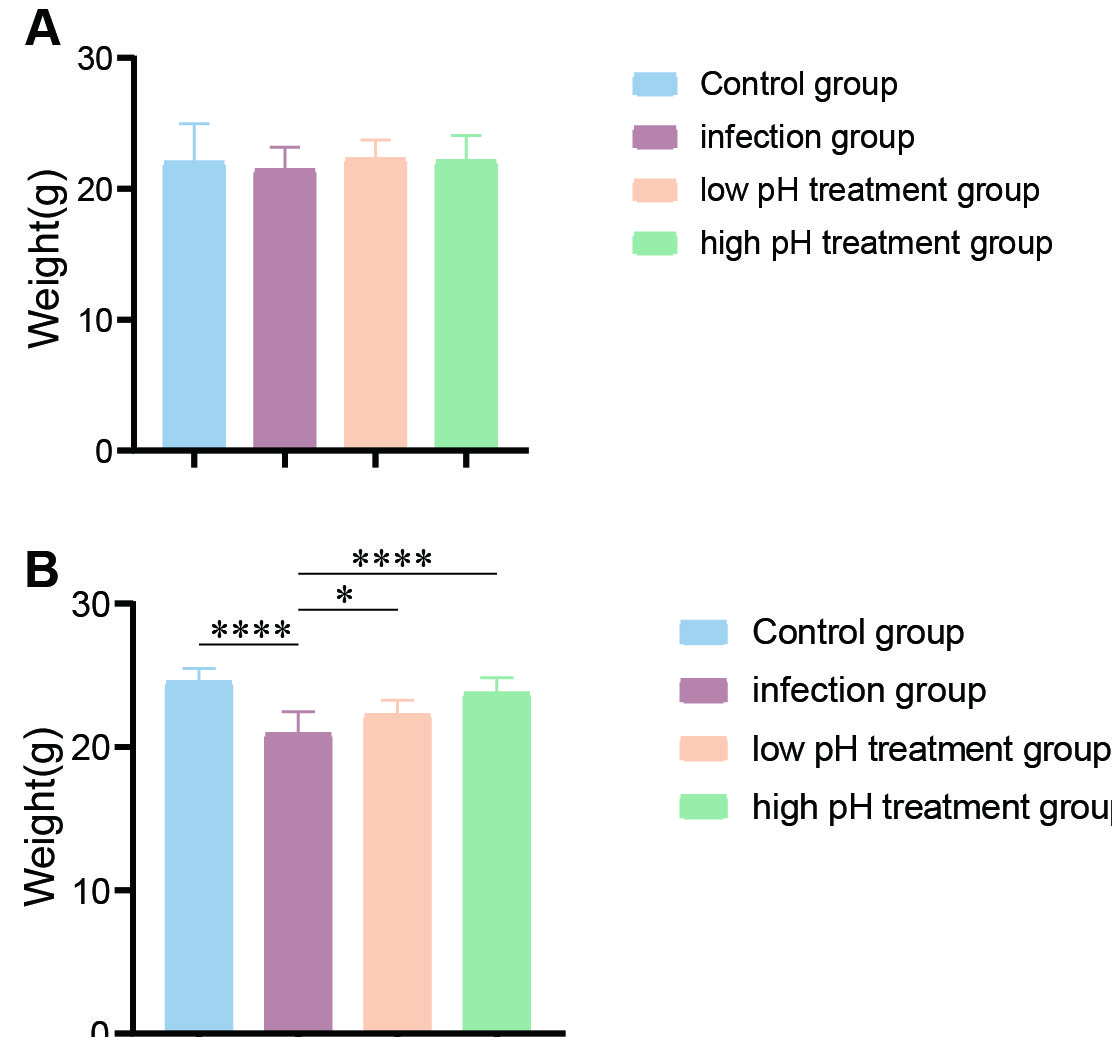

Supplement: Supplementary Figure 2 — Body weight changes in mice across different treatment groups. Body weight trends of mice in each group during the experiment. *p < 0.05, ****p < 0.0001. The infection group was inoculated with 1 × 10^8^ colony-forming units (CFU) per mouse, with n = 10 per group. [file Image2.jpeg]
